# Annotations

**Published:** 1910-01-22

**Authors:** 


### Annotations.

### The Late Dr. Skene Keith.

Known formerly as a prominent Edinburgh physician and a popular medical writer of some note, Dr. Keith died at an advanced age on January 12 last. A son of the manse, he was educated at Aberdeen and Edinburgh, coming at the latter city into close connection with Simpson, the discoverer of chloroform. Working on the apparently unpromising line of a treatment which restricted diet, and, in those days at least, avoided drugs to the point of therapeutic nihilism, he nevertheless obtained plenty of patients. Twenty years ago he retired, wrote his well-known "Plea for a Simpler Life" and "Fads of an Old Physician," which both ran into many editions, and further indulged his taste for travelling. Burmah he visited when upwards of eighty years of age; indeed, whatever share his opinions on moderation in diet may have had in the matter, he could certainly show in his own person a striking example of combined longevity and vigour. His books brought him many correspondents in all parts of the world, and his attractive personality and his culture very many friends, although naturally he had long outlived the majority of his contemporaries.

### Desirable Prohibition."

At its sitting of October 26, 1909, the French Senate adopted a resolution conceived in the following terms:—"Article 1: The selling, the offering, and exposing for sale and the importation of infants' feeding-bottles with tubes are forbidden. Article 2: The inspectors of pharmacies and the authorities cited in Article 2 of the decree of July 31, 1906, shall be entrusted with the duties of seeing to the application of this present law, which shall only come into force three months after its promulgation. Article 3: All infraction of the provisions of this present law shall be punished by a fine of 25 to 100 francs, and in the case of a second offence by imprisonment for eight days to one month. Article 463 of the penal code is applicable. In all cases the tribunals shall have power to order the confiscation of bottles seized when the law is contravened." In view of the difficulty which exists in efficiently cleansing feeding-bottles with long tubes, it would be well if similar resolutions were adopted in this country.

### "What Happens in Holloway."

There was an old custom—graceful and, in the esoteric sense of the word, "sportsmanlike"—by which martyrs or criminals would exchange preliminary courtesies with their actual torturers or executioners, the fact being thus recognised that the latter acted as mere instruments of the will of others. The spirit of this ceremony is, it must be feared, yet unreached by the body now known as Suffragists. In a recent number of a periodical concretely named *The Vote*, an article appears signed "One who is undergoing forcible feeding"; and this heroic or recalcitrant lady says that when "at last" the feeding-tube is in position the doctor nods complacently. This strikes us as a rather queer adverb to use under the circumstances, since lately prison medical officers unlucky enough to be stationed in districts visited by prominent politicians have had a great deal of anxious work added to their duties with no good prospect of extra remuneration. We have given the alternatives of heroism or rebellious obstinacy, but it may be doubted whether mediæval martyrs would have made much of a trifling epistaxis, and no good evidence exists that even this is a common occurrence. It is interesting, too, to observe that in this journal are advertised scent bottles, tea rooms, false teeth, millinery, and manicuring. Another article, which has a certain medical smack, but the name of whose author does not figure in the current Medical Directory, contains sarcasm at the expense of politicians who put the responsibility on their medical officers; and here, perhaps, a good few outside the ranks of partisans of woman suffrage will be found to agree.

### Kissing the Book.

The Oaths Act came into operation with the New Year, and it is gratifying to find that witnesses all over the country are taking advantage of the new method of testifying which this little measure renders legal. In some courts, where the justices are progressives in the true sense of the term, the Scotch form of taking the oath has been much honoured, and the old uncleanly way of kissing a grimy Testament or Bible has been left to those only who desired to adhere to it. Thus in the City and Southwark Coroner's Court, Dr. Waldo has, for some years past, intimated his willingness to administer the oath in the Scotch form and now that the new Act is operative he has been at some pains to explain its simple provisions to jurymen and witnesses. The oath to be taken by coroners' juries is a long-winded statement of some 82 words, and therefore not so simple as the old version, but it is gratifying to notice that so far the Act has worked admirably; no objection to the new form has been taken by jurors or witnesses, and the ceremony of administration goes without a hitch and, as Dr. Waldo states, "with greater dignity and impressiveness than one would have expected." Every medical man will be whole-heartedly in favour of the reform. The old method had little, if anything, to commend it, and the dangers which lay in kissing the book, especially such a book as was usually provided for Metropolitan witnesses, were not entirely fantastical and unfounded.

